# A Decade of Close-to-Nature Transformation Alters Species Composition and Increases Plant Community Diversity in Two Coniferous Plantations

**DOI:** 10.3389/fpls.2020.01141

**Published:** 2020-07-24

**Authors:** Angang Ming, Yujing Yang, Shirong Liu, You Nong, Yi Tao, Ji Zeng, Ning An, Changhai Niu, Zhang Zhao, Hongyan Jia, Daoxiong Cai

**Affiliations:** ^1^ Experimental Center of Tropical Forestry, Chinese Academy of Forestry, Guangxi Youyiguan Forest Ecosystem Research Station, Pingxiang, China; ^2^ Hubei Key Laboratory of Regional Development and Environmental Response, Faculty of Resources and Environmental Sciences, Hubei University, Wuhan, China; ^3^ Key Laboratory of Forest Ecology and Environment, State Forestry Administration, Institute of Forest Ecology, Environment and Protection, Chinese Academy of Forestry, Beijing, China

**Keywords:** close-to-nature transformation, community structure, *Cunninghamia lanceolata*, *Pinus massoniana*, plant diversity, plantation, sustainable forest management

## Abstract

Close-to-nature transformation silviculture is a promising approach to meet the criteria for sustainable forestry. To explore the effects of close-to-nature transformation on community structure and plant diversity in *Pinus massoniana* and *Cunninghamia lanceolata*s pure plantations, four stands were selected, including close-to-nature transformed stand of *P. massoniana* (PCN) and its unimproved pure stand (PCK), and close-to-nature transformed stand of *C. lanceolata* (CCN) and its unimproved pure stand (CCK). Plant diversity and community structure in the four stands were investigated before and after a decade of close-to-nature transformation. After the close-to-nature transformation, the plant diversity and community structure were significantly altered. Compared with control stands, the transformation increased the species richness and diversity of the tree layer and the whole community, while did not significantly affected the shrub and herb diversity. The species richness in the tree layer in the *P.* *massoniana* and *C.* *lanceolata* plantations was 2.1 and 2.8 times that of their corresponding control. Species composition and important value of each species were altered in the tree, shrub and herb layers. The close-to-natural transformation lowered the community dominance and the important value of *P. massoniana* and *C. lanceolate*. The advantage position of single species in the community was weakened by the forest transformation. The plant community became diversified and uniformly distributed. The enhanced community species diversity was derived from the increase in the tree diversity. These results indicated that close-to-nature transformation increased the forest plant diversity and optimized the community structure. The close-to-nature transformation plays a positive role in coniferous plantation ecosystem structure.

## Introduction

As an important component of world forest resources, plantation plays a critical role in sustainable forest management. China owns the largest plantation area in the world, 63% of which is located in southern subtropical regions (Liu et al., 2014). Coniferous plantations account for 72% of the plantations in this region, where short-rotation (<21a) industrial timber forests such as *Pinus massoniana* (21a), *Cunninghamia lanceolata* (16a), and *Eucalyptus* spp. (5a) were dominated (Cai et al., 2007). Unreasonable forest management including short-rotations of same species, excessive and premature logging, clear cutting and prescribed burning not only result in the productivity reduction and nutrient deficiency, but also cause frequent occurrence of pests and diseases and biodiversity decline (Tian et al., 2011; Zhang and Wang, 2012). Planting indigenous broad-leaved tree species in monoculture forests can increase species diversity, maintain soil fertility, enhance ecosystem stability (Carnevale and Montagnini, 2002), and increase economics (Knoke et al., 2008). Therefore, the establishment of coniferous broad-leaved mixed forest through close-to-nature transformation is gradually becoming a promising silvicultural approach to replace the large-area coniferous plantations (Emborg et al., 2000; Vesterdal et al., 2008).

The close-to-nature transformation of coniferous plantations is a number of management measures that can promote natural regeneration of seedlings and saplings and save the forest management cost through community structure adjustment (Wang and Liu, 2011; Brang et al., 2014). Following the principle of close-to-nature transformation, the tree density of the original forest is firstly reduced by thinning, and other tree species are then underplanted to transform the pure even-aged coniferous forest into uneven-aged coniferous broad-leaved mixed forest (Schütz, 1999). The close-to-nature transformation has become an approach to meet the criteria for sustainable forestry (Larsen and Nielsen, 2007) and has a history of more than 100 years (Schütz, 1999; Brang et al., 2014). The species diversity and ecosystem services in the coniferous plantations have been significantly increased by this forest management approach (Knoke et al., 2008; Luo et al., 2013; Ming et al., 2019).

The forest community structure composition plays a vital role in maintaining the structure and function of the entire forest ecosystem (Navarro et al., 2018). A forest ecosystem with a complex composition of tree species is usually stable, and thus has a high capability to combat disturbance and disease. Only healthy and stable stands can realize sustainable development. Thus, the plant composition and diversity, and structural complexity of forest community are important indicators to determine the effectiveness of sustainable forest management (Goosem et al., 2016; Douda et al., 2017).

The forest community species composition, structure, and function can be significantly affected by forest management (Montoro Girona et al., 2016; Depauw et al., 2019; Orczewska et al., 2019). The close-to-nature transformation doubtless alters the plant diversity of the tree layer, because some tree species (usually shade tolerant) are underplanted during the forest transformation. Stand density adjustment also affects plant composition and diversity (Rossman et al., 2018). Although there have been studies on the effects of selective cutting and forest conversion on understory plant composition and diversity (Heinrichs and Schmidt, 2009; Verstraeten et al., 2013), the long-term effects of close-to-nature transformation on the plant composition of the understory vegetation remains unclear. Since the plant community succession is a long-term process which is directed by the community composition, there is a need to explore the long-term effects of the promising silvicultural approach, close-to-nature transformation, on plant community composition and diversity. This will help understand the impact of close-to-nature transformation on forest sustainability.

Therefore, we selected two typical coniferous plantations in subtropical China, *P. massoniana* and *C. lanceolata*, to explore the long-term effects of close-to-nature transformation on the structure and diversity of forest plant community. The forest transformation has been conducted for more than 10 years. Since soil properties can affect plant growth and diversity, we also measured the soil basic physicochemical properties to explore how the forest transformation affects plant diversity by altering soil properties. We hypothesized that the long-term close-to-nature transformation shifted the species composition, and increased species diversity of the forest plant community. This study is aiming at providing a scientific basis for sustainable forest management and multi-objective silviculture of coniferous plantations in southern subtropical regions.

## Materials and Methods

### Study Site

This study was conducted at the Guangxi Youyiguan Forest Ecosystem Research Station, the Experimental Center of Tropical Forestry, Chinese Academy of Forestry (22°10’ N, 106°50’ E, Pingxiang, Guangxi, China). It is one of the forest ecology research stations under the jurisdiction of the State Forestry and Grassland Administration. The site has a subtropical monsoon climate, with a semi-humid climate and obvious dry and wet seasons. The annual sunshine duration is 1200–1600 h. Precipitation is abundant, with an annual average of 1200–1500 mm, mainly from April to September. The annual evaporation is 1200–1400 mm, the relative humidity is 80%–84%, and the average annual temperature is 20.5–21.7°C. The study area is dominated by monocultures of *P. massoniana*, *C. lanceolatam*, indigenous broad-leaved plantations of *Erythrophleum fordii*, *Castanopsis hystrix*, *Mytilaria laosensis*, and *Michelia macclurei*, as well as some mixed plantations. The main types of landforms are low hills and hills. The soil is mainly composed of laterite and red soil based on the Chinese soil classification; this is classified as a ferralsol in the World Reference Base for Soil Resources. Soil depth is generally greater than 80 cm. Subtropical evergreen broad-leaved forests comprise the local vegetation.

### Experimental Design

**Figure 1:**
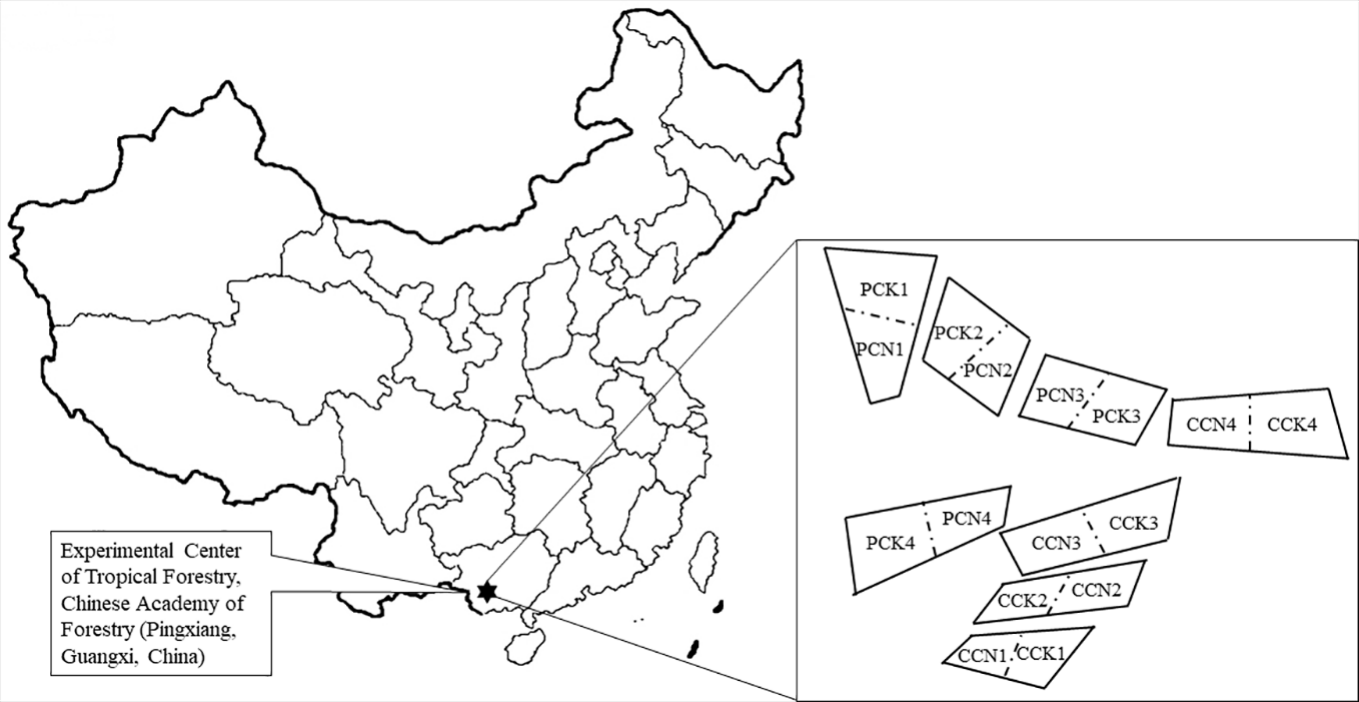
A schematic plot of studying site and experimental design. PCK, PCN, CCK, and CCN represent the pure and close-to-nature managed *P. massoniana* plantation and the pure and close-to-nature managed *C. lanceolata* plantation, respectively. Numbers from 1 to 4 represent plot replications. Each plot was 20m×20m.

This study followed our previous experiment (Ming et al., 2018; Ming et al., 2019). In brief, a single-factor and two-level stochastic block design was used. There were four blocks representing four replicates. Four forest types were set up in each block: The close-to-nature *P. massoniana* plantation (PCN), the unimproved *P. massoniana* pure plantation (PCK), the close-to-nature *C. lanceolata* plantation (CCN), and the unimproved *C. lanceolata* pure plantation (CCK). There were thus a total of sixteen 20m×20m experimental plots (**Figure 1**). During the close-to-nature transformation, the forest density was reduced from 1200 trees ha^-1^ to 450 trees ha^-1^ by intensity thinning from below, and *Quercus griffithii* and *Erythrophleum fordii* were underplanted with both density of 375 trees ha^-1^. Therefore, after the close-to-nature transformation, all the stands had a density of 1200 trees ha^-1^.

### Quadrat Survey

In each 20m×20m plot, the plant height and diameter breast height (DBH) of each tree were measured using ultrasonic-based device Vertex IV (Haglöf Sweden AB) and tapeline, to determine the plant composition and diversity of the tree layer. Four 5m×5m quadrat was randomly set in each plot to investigate the plant composition and diversity of the shrub and herb layers. Woody plants with height < 5m and all lianas belong to the shrub layer. A HemiView Digital Canopy Analysis System (Delta-T Devices Ltd., Cambridge, England) was used to determine the forest canopy density at five randomly selected points in each plot. Also, six 1m×1m litterfall collectors were randomly arranged in each plot to collect litterfall. At the end of each month, the litterfall was brought to laboratory to be weighted after oven-dried at 65°C. The weights of the litterfall were then used to calculate the annual litterfall yield. The quadrat survey was made before the close-to-nature transformation in 2007 and a decade later in 2018. Before the forest transformation, the canopy density of the *P. massoniana* and *C. lanceolata* plantation was 0.94 ± 0.04 and 0.85 ± 0.09, respectively, and the current status of the stands was shown in **Table 1**.

**Table 1:** Basic characteristics of the four stands in August 2018.

Plantation types	PCK	PCN	CCK	CCN	Treatment	Species	Treatment× Species
Stand age	25	25	25	25	—	—	—
Slope aspect	Northwest	Northwest	Southwest	Southwest	—	—	—
Slope (°)	21.3 ± 3.6	22.4 ± 4.1	24.6 ± 2.6	23.1 ± 2.9	ns	ns	ns
Canopy density	0.71 ± 0.09	0.88 ± 0.03	0.78 ± 0.09	0.79 ± 0.11	ns	ns	0.001
Basal area (m^2^ ha^-1^)	51.81 ± 0.21	61.43 ± 0.90	31.98 ± 2.18	50.82 ± 1.21	ns	ns	ns
Litterfall (t ha^-1^ yr^-1^)	10.23 ± 0.94	10.84 ± 0.49	9.02 ± 0.19	9.54 ± 0.34	ns	0.019	ns

Data are shown as means ± standard errors (n=4). The results (p values) from ANOVA are shown in the right panel of the table. PCK, PCN, CCK, and CCN represent the pure and close-to-nature managed P. massoniana plantation and the pure and close-to-nature managed C. lanceolata plantation, respectively. ns, non-significant (p > 0.05).

### Measurements and Data Compilation

We used important value (IV) to reflect the degree of relative dominance of each species within the plant community. Along with the plant diversity indexes, the plant composition and structure of the tree, shrub and herb layers were described using the following formulas (Shaheen et al., 2012):

(1) IV in tree layer=(relative frequency + relative density + relative significance)/3

(2) IV in shrub and herb layer=(relative frequency + relative density + relative coverage)/3

(3) $$\text{Margalef}\text{index}=\frac{S-1}{lnN}$$

(4) $$\text{Shannon-Wiener index}=\Sigma_{i=1}^{s}\;pilnpi$$

(5) $$\text{Simpson index}=1-\Sigma_{i=1}^{s}\;(pipi)$$

(6) $$\text{Pielou index}=\frac{H^{\prime }}{lnS}$$

(7) $$\text{Ecological}{\text{dominance = Σ}}_{i=1}^{\text{s}}\;ni(ni-1)/N(N-1)$$

Where *S* is species number, *N* is total number of individuals, *pi* is the proportion of individuals of species *i*, *H’* is the Shannon-Wiener index, and *ni* is the number of individuals in species *i*.

To explore the causes of the changes in plant diversity, we also determined soil properties. Twelve soil samples at a depth of 0–10 cm were randomly collected using a stainless steel soil auger with an inner diameter of 8.7 cm. These samples were placed in mixed sample bags for preservation. The soil samples were then taken back to the laboratory to remove coarse roots, rubble, and other impurities using a 2 mm aperture screen and air dried for physicochemical analysis. Soil water content, bulk density, and porosity were measured using the volumetric ring during field sampling. Soil pH value was measured by glass electrode method (1 mol L^-1^ KCl solution extraction). Soil organic C was measured by potassium dichromate external heating method. Soil total N was measured by Kjeldahl method. Soil NH_4_^+^-N and NO_3_^–^N were measured by spectrophotometry. Soil available N was analyzed through quantification of alkali-hydrolysable N in a Conway diffusion unit with Devarda’s alloy in the outer chamber and boric acid-indicator solution in the inner chamber (Shen et al., 2004). Soil total P and total K were measured by molybdenum antimony colorimetry and flame photometry, after NaOH alkaline melting, respectively. Soil available P and K were measured by double acid infusion—molybdenum antimony colorimetry and ammonium acetate—flame photometer method, respectively (Pansu and Gautheyrou, 2006).

### Statistical Analysis

A mixed-effect analysis of variance (ANOVA) was performed to analyze the effects of close-to-nature transformation treatment, original plant species (i.e. *P. massoniana* and *C. lanceolata*) and their interaction on the community diversity and structure of the tree, shrub and herb layer, as well as soil physicochemical properties. In the ANOVA model, the treatment and species were set as fixed and random effect, respectively. Pearson correlations were adopted to examine the correlations between plant diversity and environmental factors in the stands. All the statistical analyses were performed using SPSS 19.0 (SPSS, Inc, Chicago, IL).

## Results

### The Effects of Close-to-Nature Transformation on Plant Community Structure

**Figure 2:**
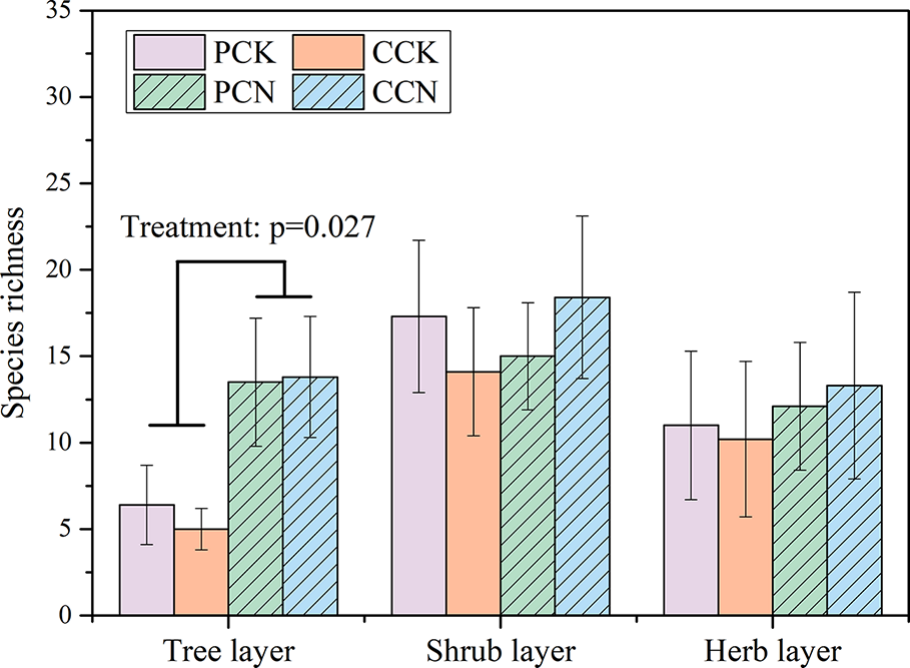
Plant species richness in the four stands. Data are shown as means ± standard errors (n=4). Significant differences between the control and close-to-nature managed plantations are indicated by the p values from the results of ANOVA (p < 0.05). PCK, PCN, CCK, and CCN represent the pure and close-to-nature managed *P. massoniana* plantation and the pure and close-to-nature managed *C. lanceolata* plantation, respectively.

**Figure 3:**
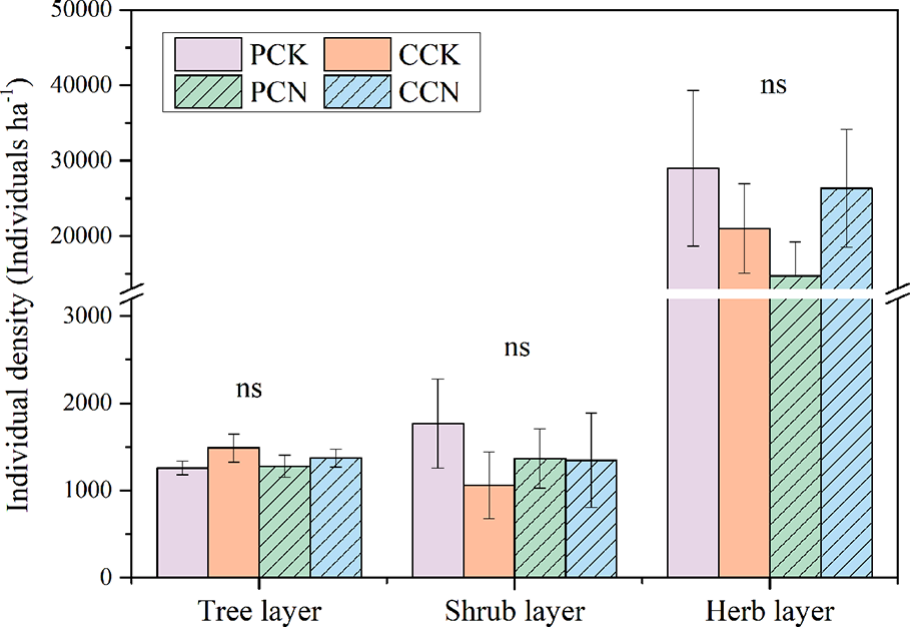
Plant individual density in the four stands. The density in the tree and shrub layer refers to the numbers of stems per ha, and that in the herb layer refers to the number of herb clusters per ha. Data are shown as means ± standard errors (n=4). Ns indicates the differences between the control and close-to-nature managed plantations were not significant according to the results of ANOVA (p > 0.05). PCK, PCN, CCK, and CCN represent the pure and close-to-nature managed *P. massoniana* plantation and the pure and close-to-nature managed *C. lanceolata* plantation, respectively.

The close-to-nature transformation altered the species richness in the tree layer, but not in the shrub or herb layer (**Figure 2**). The tree species numbers in the *P. massoniana* and *C. lanceolata* transformed forests were 2.1 and 2.8 times than those of their control stands, respectively. In contrast, the close-to-nature transformation did not significantly alter the individual densities in the tree, shrub and herb layers (**Figure 3**).

In 2018 that after a decade of forest transformation, a total of 23 species were found in the tree layer of the four stands, belonging to 23 genera in 20 families (**Table 2**). There were 7 and 15 species in the *P.* *massoniana* control and transformed stands, respectively, whereas there were five and 14 species in the *C. lanceolata* control and transformed stands, respectively. Compared with the control, indigenous tree species including *Ficus esquiroliana*, *Schefflera* *minutistellata*, *Trema* *cannabina* *Lour*. var. *dielsiana*, and *Canthium* *horridum* were added in the *P.* *massoniana* transformed stands, whereas *Melia* *azedarach*, *Evodia* *lepta*, and *Sapium* *discolor* were added in the *C. lanceolata* transformed stands. According to the important value, besides *P. massoniana*, the PCK stand was dominated by *Schima* *wallichii*, whereas PCN was dominated by *Q. griffithii* and *E. fordii*. For the *C. lanceolata* stands, the control stand was dominated by *C. lanceolata* and *Aporusa villosa*, whereas the transformed stand was dominated by *C. lanceolata, Q. griffithii* and *E. fordii*. As compared with the control, the close-to-nature transformation significantly decreased the important values of *P*. *massoniana* and *C.* *lanceolata* by 32.78% and 36.39% in the *P*. *massoniana* and *C.* *lanceolata* stands, respectively. The reduced important values were basically replaced by the underplanted *Q. griffithii* and *E. fordii*.

**Table 2:** Species composition and important value in the tree layer in the four stands.

Species name	Forest type			
	PCK	PCN	CCK	CCN
*Pinus massoniana*	61.47	41.32		
*Cunninghamia lanceolata*			63.86	40.62
*Quercus griffithii*		16.00		14.79
*Erythrophleum fordii*		12.72		12.54
*Styrax tonkinensis*	7.09	7.07		
*Ficus esquiroliana*		3.96		3.68
*Schefflera minutistellata*		2.93		4.02
*Syzygium hainanense*		2.65		
*Trema cannabina Lour*. var. dielsiana		2.59		4.08
*Canthium horridum*		2.25		1.86
*Schima wallichii*	10.03	2.20		2.00
*Cratoxylum cochinchinense*		2.14		
*Litsea cubeba *		2.09		2.57
*Aporusa villosa*	7.15	2.07	12.59	1.94
*Evodia lepta*				3.73
*Melia azedarach*				1.99
*Saurauia tristyla*			6.95	1.80
*Machilus chinensis*	5.23			
*Celtis timorensis*	4.78			
*Schefflera minutistellata*	4.25			
*Alangium kwangsiense*			8.77	
*Microcos paniculata*			7.84	
*Sapium discolor*				0.61

PCK, PCN, CCK, and CCN represent the pure and close-to-nature managed P. massoniana plantation and the pure and close-to-nature managed C. lanceolata plantation, respectively.

In the shrub layer, a total of 33 species were found in the four stands, belonging to 28 genera in 16 families (**Table 3**). Compared with the controlled stands, the close-to-nature transformation removed heliophilous shrubs, including *Clerodendrum cyrtophyllum*, *Aralia* *chinensis*, *Sapium* *discolor*, and *Phyllanthus* *emblica* in the *P.* *massoniana* stand, while introduced heliophilous native species, including *Ficus hispida*, *Melastoma sanguineum* and *P. emblica.*

**Table 3:** Species composition and important value in the shrub layer in the four stands.

Species name	Forest type			
	PCK	PCN	CCK	CCN
*Evodia lepta*	8.23	10.48	23.28	4.71
*Ficus esquiroliana*	3.18	9.76	14.39	2.16
*Aporusa villosa*	5.90	4.92	11.44	2.29
*Clerodendrum cyrtophyllum*	6.63		11.11	12.55
*Litsea cubeba *			5.48	14.31
*Saurauia tristyla*			6.01	10.84
*Schefflera minutistellata*	8.22	3.68		2.55
*Litsea glutinosa*		2.05		2.29
*Aralia chinensis*	1.42			4.70
*Mallotus philippensis*	4.42	2.05	2.72	7.86
*Ficus hirta *	7.12	11.30		3.80
*Ficus hispida*				3.15
*Liquidambar formosana*	2.24			
*Callicarpa rubella*			3.07	2.55
*Clerodendrum canescens*	2.47			
*Maesa perlarius*	8.16	4.49	2.55	1.79
*Cipadessa baccifera*			4.10	3.52
*Psychotria rubra*	4.03	2.67		
*Glochidion eriocarpum*		4.51		
*Actinodaphne pilosa*	7.03	2.67		
*Melastoma sanguineum*				2.42
*Mallotus barbatus*				6.00
*Vernicia montana*	2.70			
*Viburnum fordiae*	2.72			
*Toxicodendron vernicifluum*	2.24	9.61	6.82	5.81
*Sapium discolor*	1.51			
*Helicteres angustifolia*			3.24	
*Wendlandia uvariifolia*	5.26			
*Alangium faberi*		7.52		
*Phyllanthus emblica*	2.24			2.91
*Macaranga denticulata*	2.24	9.00	5.79	
*Canthium horridum*	12.03	15.27		
*Rhus chinensis*				3.78

PCK, PCN, CCK, and CCN represent the pure and close-to-nature managed P. massoniana plantation and the pure and close-to-nature managed C. lanceolata plantation, respectively.

In the herb layer of the four stands, there were a total of 17 species belonging to 16 genera in 10 families, of which 11, 11, 8, and 13 in the PCK, PCN, CCK and CCN stands, respectively (**Table 4**). Gramineous grasses including *Lophatherum gracile, Cyrtococcum patens*, and *Microstegium vagans*, and ferns including *Pronephrium lakhimpurense*, *Cyclosorus parasiticus* and *Dicranopteris dichotoma* were added in the transformed forests than control. However, heliophilous plants like *Dianella* *ensifolia* and *Lindsaea* *orbiculata* were removed. The forest transformation also affected the species important values in the shrub and herb layers.

**Table 4:** Species composition and important value in the herb layer in the four stands.

Species name	Forest type			
	PCK	PCN	CCK	CCN
*Pteris semipinnata*		15.03	19.98	3.83
*Lophatherum gracile*		8.78		3.44
*Cyrtococcum patens*	36.21	11.21		5.94
*Pronephrium lakhimpurense*	9.96			4.40
*Cyclosorus parasiticus*				3.01
*Cibotium barometz*		4.04	17.16	4.95
*Pteris fauriei*			1.91	
*Microstegium vagans*	15.03	5.00		25.35
*Dicranopteris dichotoma*	10.59	11.45		9.04
*Dianella ensifolia*	2.39			
*Alpinia japonica*	5.97	6.14	3.35	1.02
*Adiantum flabellulatum*	8.08	14.21	5.07	2.63
*Lindsaea orbiculata*	2.20			
*Blechnum orientale*	1.43	15.28	45.52	21.48
*Miscanthus floridulus*	4.53	4.97	1.96	6.54
*Schizoloma heterophyllum*	3.61	3.89		
*Thysanolaena maxima*			5.05	8.37

PCK, PCN, CCK, and CCN represent the pure and close-to-nature managed P. massoniana plantation and the pure and close-to-nature managed C. lanceolata plantation, respectively.

### The Effects of Close-to-Nature Transformation on Plant Diversity and Evenness

**Figure 4:**
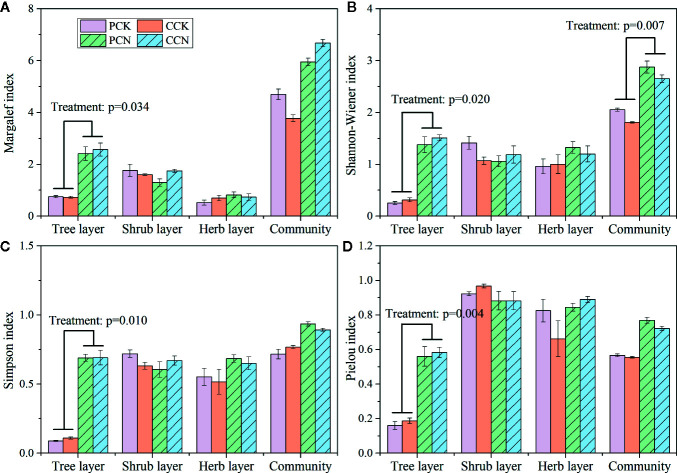
Margalef index **(A)**, Shannon-Wiener index **(B)**, Simpson index **(C)**, and Pielou index **(D)** in the four stands. Data are shown as means ± standard errors (n=4). Significant differences between the control and close-to-nature managed plantations are indicated by the p values from the results of ANOVA (p < 0.05). PCK, PCN, CCK, and CCN represent the pure and close-to-nature managed *P. massoniana* plantation and the pure and close-to-nature managed *C. lanceolata* plantation, respectively.

The close-to-nature transformation significantly increased the Margalef, Shannon-Wiener, Simpson, and Pielou indexes in the tree layer (**Figure 4**). In the *P.* *massoniana* and *C.* *lanceolata* plantations, the Shannon-Wiener index of the tree layer in the transformed stand was 5.5 and 5.3 times, Simpson index was 7.8 and 6.3 times, and Pielou index was 3.4 and 3.3 times of the control forest, respectively. However, the close-to-nature transformation did not significantly affect the plant diversity indexes of the shrub and herb layers. Besides, the forest transformation significantly increased the community diversity of the whole plantation.

**Figure 5:**
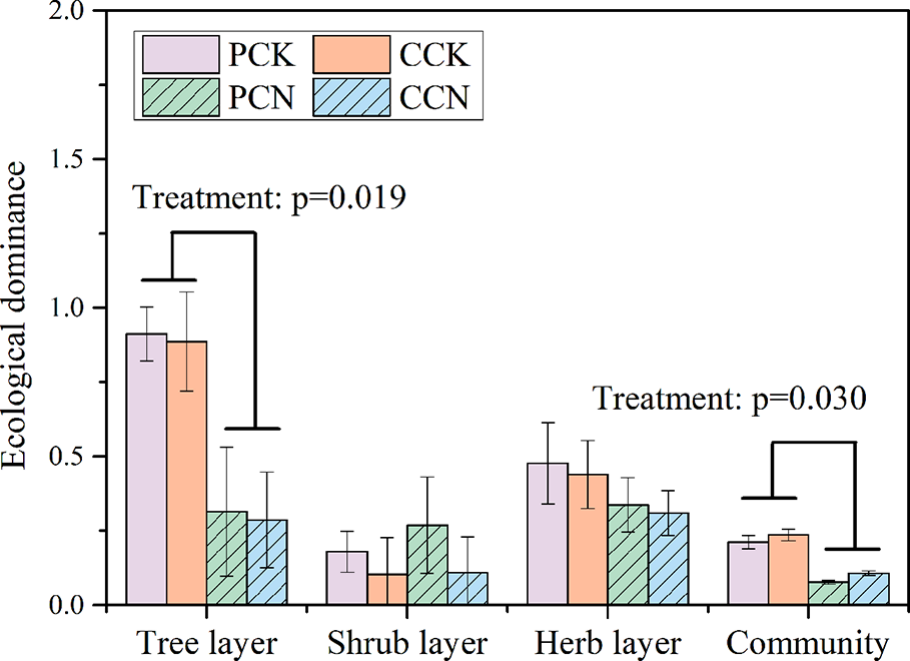
Ecological dominance of plants in the four stands. Data are shown as means ± standard errors (n=4). Significant differences between the control and close-to-nature managed plantations are indicated by the p values from the results of ANOVA (p < 0.05). PCK, PCN, CCK, and CCN represent the pure and close-to-nature managed *P. massoniana* plantation and the pure and close-to-nature managed *C. lanceolata* plantation, respectively.

The close-to-nature transformation reduced the ecological dominance of the tree layer, whereas it did not affect that of shrub and herb layer (**Figure 5**). Overall, the transformation negatively affected the community dominance in both plantations.

### The Main Influencing Factors on Community Structure and Diversity

As shown from the survey in 2018, the forest transformation did not affect the canopy density, basal area, or annual litterfall yield (**Table 1**). Among all the measured soil properties, the forest transformation only significantly affected soil organic C, total N, and available K contents (**Table 5**). Compared to the control, the transformation increased the contents of soil organic C and total N by 17.4% and 6.8%, respectively, whereas reduced the soil available K content by 20.6%.

**Table 5:** Soil physicochemical properties in the four stands.

Variables	PCK	PCN	CCK	CCN	Treatment	Species	Treatment× Species
Water content (%)	18.49 ± 2.49	19.66 ± 1.69	16.63 ± 0.86	16.65 ± 2.94	ns	ns	ns
Bulk density (g cm^-3^)	1.07 ± 0.14	1.12 ± 0.09	1.28 ± 0.06	1.34 ± 0.10	ns	0.025	ns
Porosity (%)	56.80 ± 2.83	56.04 ± 2.58	49.05 ± 4.99	45.17 ± 4.86	ns	ns	ns
pH (KCl)	4.18 ± 0.04	4.31 ± 0.08	4.67 ± 0.07	4.91 ± 0.20	ns	0.015	ns
Organic C (g kg^-1^)	25.99 ± 1.32	29.15 ± 2.42	17.24 ± 1.85	21.61 ± 2.58	0.018	<0.001	0.667
Total N (g kg^-1^)	2.58 ± 0.04	3.28 ± 0.12	2.29 ± 0.15	3.32 ± 0.13	0.046	<0.001	0.046
Available N (mg kg^-1^)	94.37 ± 3.94	103.32 ± 5.62	77.0 ± 9.07	96.25 ± 7.27	ns	ns	ns
Total P (g kg^-1^)	0.28 ± 0.01	0.25 ± 0.02	0.24 ± 0.03	0.21 ± 0.01	ns	ns	ns
Available P (mg kg^-1^)	1.81 ± 0.43	1.70 ± 0.28	1.15 ± 0.23	1.08 ± 0.39	ns	0.019	ns
Total K (g kg^-1^)	6.45 ± 1.06	5.80 ± 0.99	20.01 ± 5.28	16.03 ± 7.62	ns	ns	ns
Available K (mg kg^-1^)	72.98 ± 38.63	51.32 ± 21.4	125.59 ± 23.60	106.38 ± 40.42	0.038	0.015	ns
C:N	17.06 ± 0.50	15.34 ± 0.72	16.42 ± 0.14	15.16 ± 0.46	ns	ns	0.043
NH_4_^+^-N content (mg kg^-1^)	20.30 ± 2.07	26.67 ± 3.35	18.44 ± 2.17	24.56 ± 4.02	ns	ns	ns
NO_3_^–^N content (mg kg^-1^)	21.97 ± 1.83	25.00 ± 2.21	18.36 ± 2.28	24.65 ± 4.19	ns	ns	ns

Data are shown as means ± standard errors (n=4). The results (p values) from ANOVA are shown in the right panel of the table. PCK, PCN, CCK, and CCN represent the pure and close-to-nature managed P. massoniana plantation and the pure and close-to-nature managed C. lanceolata plantation, respectively. ns, non-significant (p > 0.05).

The soil organic C content significantly positively correlated with the Margalef, Shannon-Wiener, and Simpson indexes of the tree layer (**Table 6**). In the shrub layer, the stand basal area significantly negatively correlated with the Margalef, Shannon-Wiener, and Simpson indexes, and the canopy density significantly negatively correlated with the diversity indexes. Among the abiotic factors, only soil available N significantly positively correlated with the Shannon-Wiener index of the shrub layer. In the herb layer, the basal area and canopy density significantly negatively correlated with the diversity indexes, whereas soil total P significantly positively correlated with the Shannon-Wiener and Simpson indexes. However, only the soil organic C significantly correlated with the forest community diversity.

**Table 6:** Pearson’s correlations between diversity indexes and environmental variables.

Layer	Diversity indexes	Basal area	Canopy density	Soil organic C	Soil available N	Soil total P
Tree layer	Margalef index	0.410	0.445	0.518*	0.228	-0.076
	Shannon-Wiener index	0.346	0.439	0.688**	0.097	-0.035
	Simpson index	0.460	0.463	0.580*	0.143	-0.081
	Pielou index	0.217	0.445	0.316	0.137	-0.068
Shrub layer	Margalef index	-0.550*	-0.724**	-0.249	0.098	-0.131
	Shannon-Wiener index	-0.737**	-0.753**	0.166	0.529*	-0.147
	Simpson index	-0.547*	-0.892**	0.096	0.324	-0.163
	Pielou index	-0.092	-0.532*	-0.314	-0.164	0.155
Herb layer	Margalef index	-0.540*	-0.734**	-0.192	-0.187	0.455
	Shannon-Wiener index	-0.736**	-0.683*	-0.009	-0.003	0.545*
	Simpson index	-0.721**	-0.587*	0.064	0.050	0.546*
	Pielou index	-0.569**	-0.525*	0.207	0.157	0.289
Community	Margalef index	-0.410	-0.445	0.518*	0.228	-0.076
	Shannon-Wiener index	-0.346	-0.439	0.688**	0.097	-0.035
	Simpson index	-0.460	-0.463	0.580*	0.143	-0.081
	Pielou index	-0.217	-0.445	0.316	0.137	-0.068

* and ** indicated significant correlation at p < 0.05 and p < 0.01, respectively. Only the factors that were significantly correlated with the diversity index are shown.

## Discussions

### The Effects of Close-to-Nature Transformation on Community Structure and Diversity

After a decade of close-to-nature transformation, the species richness in the tree layer in the *P.* *massoniana* and *C.* *lanceolata* plantations was 2.1 and 2.8 times that of their corresponding control (**Figure 1**). Because *Q. griffithii* and *E.* *fordii* were underplanted during the forest transformation, these two broad-leaved tree species were added to the transformed stands. Meanwhile, during the initial period of the transformation, the stand density was greatly reduced by thinning, making some species in the shrub layer grow fast and grew into the tree layer. Therefore, compared with the control, the transformed forests had a higher level of tree richness.

The dominance and the important values of each species in the forest community were also affected by the close-to-nature transformation (**Tables 2**–**4**). In the transformed stands, the underplanted *Q. griffithii* and *E.* *fordii* weakened the absolute advantage of *P.* *massoniana* and *C.* *lanceolata*, reducing their important values (**Table 2**). This indicated the close-to-nature transformation diversified the structure and composition of the tree layer. This is consistent with previous studies (Brunet et al., 2010). As a result, the forest transformation reduced the community dominance index (**Figure 5**). The forest transformation processed the plant community towards structurally complex and diverse forests.

In contrast, the forest transformation did not affect the species richness and individual density of the shrub and herb layers (**Figures 2** and **3**), but altered their dominant species (**Tables 3** and **4**). This might because the changes in dominant species in the shrub and herb layers were not only influenced by light, litterfall, and soil properties, but also by the species composition in the tree layer (Small and McCarthy, 2005; Tardella et al., 2019). Newly added plant species in the tree layer reduced the light intensity of the understory. Consequently, the transformation was more conducive to the growth of shade-tolerance ferns and gramineous plants, rather than *Dianella ensifolia* and other pioneer heliophilous herbs.

Furthermore, compared with the control stands, the forest transformation enhanced the diversity indexes of the whole community and the tree layer, while no difference was observed either in the shrub or herb layer (**Figure 4**). These demonstrated that the forest transformation improved the plant diversity of the community due to the diversified species composition in the tree layer (Brunet et al., 2010). Other studies also indicated that the plant composition and structure of tree layer affect those of the forest community (Whigham, 2004; Gilliam, 2007). However, the close-to-nature transformation did not affect the shrub and herb diversity. This is contrary to some studies (Zhang et al., 2011; Luo et al., 2013). The effects of forest management on plant diversity in the understory layers showed a long-term and continuous pattern (You et al., 2016). Because only 1 year data were used, the effects of the close-to-nature transformation on species diversity in the plantations still remain uncertain.

### The Reasons for the Changed Community Structure and Diversity

The species composition and distribution of plant community are the combined result of biotic and abiotic factors. At regional scale, precipitation, temperature and elevation are the main factors affecting the vegetation growth and distribution (Siefert et al., 2012). However, in the same study area, there is no difference in the precipitation and temperature, thereby micro-environmental factors, such as soil physicochemical and biological properties, slope and its aspect may be the key factors affecting the plant composition (Pennington et al., 2017; Nie et al., 2019; Rodrigues et al., 2019). Before the forest transformation, soil properties were not significantly different in the *P. massoniana* and *C. lanceolatam* pure plantations. Therefore, the differences in soil C and N among the forests after a decade of close-to-nature transformation were derived from forest transformation. Meanwhile, community diversity was correlated with soil properties (**Table 6**), and the diversity of forest plant community is largely determined by the tree diversity (Whigham, 2004; Gilliam, 2007). Therefore, the differences in the community composition and structure between the transformed and control stands in our study were mainly caused by the changes in the soil properties and characteristics of the tree layer.

Although soil organic C was positively correlated with the plant diversity in the tree layer and community (**Table 6**), soil organic C was not the main influencing factor on plant diversity. This is because the soil organic C content was not significantly different between the stands before the forest transformation (data were not presented). The variation in soil organic C was caused by the altered community composition and diversity. Thus, the species diversity indexes of tree layer were not affected by stand canopy density, basal area or soil properties. The increased plant diversity in the tree layer was the result of planting indigenous broad-leaved species and thinning during the close-to-nature transformation. The underplanted species directly increased the species richness in the tree layer. Meanwhile, the intensity thinning provided sufficient space, especially a good light condition, for other species and seedlings. Consequently, the understory seedlings naturally grow into the tree layer, changing the plant composition and diversity of the tree layer.

In our study, the diversity indexes of the shrub and herb layers were correlated with canopy density and basal area (**Table 6**). In fact, canopy density and basal area can influence the light in the forest, which further impacts the understory plant diversity (Heinrichs and Schmidt, 2009; Tardella et al., 2019). However, in our study, the transformed stands and the controls did not differ in canopy density and basal area (**Table 1**), thus the shrub and herb diversity were not affected by the forest transformation (**Figure 4**). On the other hand, forest gaps have been created through the thinning during the forest transformation. The gaps provide habitats for plant species, and influencing the community species composition and diversity (Muscolo et al., 2014). Studies showed that the size and number of forest gap were negatively correlated with diversity index, and positively correlated with species regeneration (i.e. the density of seedlings and saplings) (Devagiri et al., 2016; Montoro Girona et al., 2018). After thinning, some forest may also experience windthrow which have a major impact on diversity creating new habitats and gaps (Peterson and Leach, 2008; Dodet et al., 2011). However, our experimental area is not in the typhoon area, and seldom exposed to typhoons or strong winds. Consequently, no windthrow were found in the experimental forests. Then we can eliminate the influence of windthrow on the plant diversity. Further studies on the dynamics of forest gaps generated by thinning will make a deep understanding on the changes in the plant diversity. To strengthen the studies on the effects of different thinning intensities and gap sizes on the species diversity may further reveal the maintenance and variation mechanisms of plant diversity. Additionally, such variations in plant diversity on the ecosystem function can also be a future research direction.

## Conclusions

The decade of close-to-nature transformation significantly affected the community structure of *P. massoniana* and *C. lanceolata* plantations, increasing the species diversity in the tree layer and community. The forest transformation reduced the dominance of *P. massoniana* and *C. lanceolata*, and diversifying and uniformly distributing the plant species in the community. The altered species diversity in the tree layer directly led to the changes in the community species diversity. The altered tree species composition and increased diversity were the combined results of forest thinning and indigenous species underplanting during the close-to-nature transformation. The forest transformation increased the plant diversity and optimized the community structure of *P. massoniana* and *C. lanceolata* monoculture plantations. The close-to-nature transformation can be a promising approach to promote the structure of the coniferous plantation ecosystem.

## Data Availability Statement

The datasets generated for this study are available on request to the corresponding author.

## Author Contributions

AM collected data and drafted the manuscript. YY revised the manuscript and participated in analyzing the experiment data. SL and DC conceived and designed the work. YN, YT, JZ, NA, CN, ZZ, and HJ participated in collecting the experiment data. All authors contributed to the article and approved the submitted version.

## Funding

This study was supported by the 13th Five-Year National Key Technology R&D Program (No. 2017YFD0600304), and Guangxi forestry science and technology projects [Document of Guangxi forestry department (2016) No.37].

## Conflict of Interest

The authors declare that the research was conducted in the absence of any commercial or financial relationships that could be construed as a potential conflict of interest.
